# A New Route for High-Purity Organic Materials: High-Pressure-Ramp-Induced Ultrafast Polymerization of 2-(Hydroxyethyl)Methacrylate

**DOI:** 10.1038/srep18244

**Published:** 2015-12-16

**Authors:** E. Evlyukhin, L. Museur, M. Traore, C. Perruchot, A. Zerr, A. Kanaev

**Affiliations:** 1 Laboratoire de Physique des Lasers - LPL, CNRS, Université Paris 13, Sorbonne Paris Cité, 93430 Villetaneuse, France; 2 Laboratoire des Sciences des Procédés et des Matériaux - LSPM, CNRS, Université Paris 13, Sorbonne Paris Cité, 93430 Villetaneuse, France; 3 Laboratoire Interfaces Traitements Organisation et Dynamique des Systèmes - ITODYS CNRS, Université Paris Diderot Paris 7, Sorbonne Paris Cité, 75205 Paris Cedex 13, France

## Abstract

The synthesis of highly biocompatible polymers is important for modern biotechnologies and medicine. Here, we report a unique process based on a two-step high-pressure ramp (HPR) for the ultrafast and efficient bulk polymerization of 2-(hydroxyethyl)methacrylate (HEMA) at room temperature without photo- and thermal activation or addition of initiator. The HEMA monomers are first activated during the compression step but their reactivity is hindered by the dense glass-like environment. The rapid polymerization occurs in only the second step upon decompression to the liquid state. The conversion yield was found to exceed 90% in the recovered samples. The gel permeation chromatography evidences the overriding role of HEMA_2_^••^ biradicals in the polymerization mechanism. The HPR process extends the application field of HP-induced polymerization, beyond the family of crystallized monomers considered up today. It is also an appealing alternative to typical photo- or thermal activation, allowing the efficient synthesis of highly pure organic materials.

High-pressure (HP) technologies are a very important tool in materials science and are widely used to transform an initial material into a new material that exhibits properties of high technological interest. The synthesis of diamond from graphite under high pressure is one of the most striking examples in this field^1^. Recently, the increasing attention to environmental issues and the growing demand for the development of green chemistry processes^2^ has opened new fields of applications for HP technologies^3^. In this perspective one of the most important benefits of HP chemistry is the possibility of initiating chemical reactions without the use of potentially toxic solvents, catalysts or radical initiators^4^,^5^,^6^. High-pressure science is also important in fundamental condensed matter physics and chemistry. Essentially, HP-induced chemical reactions are governed by two factors that may compete under certain conditions. First, an increased density leads to large modifications in the intermolecular interactions and electronic structure, making the system thermodynamically unstable. Second, the stress induced by the HP results in significant potential energy barriers, which can lead to selectivity in the reaction pathways. For example, in molecular crystalline solids, the reactant orientations can promote or prevent a specific reaction channel according to the so-called topochemical principle. Therefore, although the energy added to the system by compression may be sufficient to initiate the reaction, the constraints induced by the dense environment can prevent molecular rearrangements along the reaction coordinate and even impede the reaction. Examples of irreversible or reversible HP-induced chemical reactions have been reported in several reviews^7^,^8^,^9^.

Among the HP-induced chemical reactions, polymerization attracts significant interest due to the possibility of obtaining pure polymers that are free of radical-generating additives. In the past decade, the HP polymerization of small, unsaturated hydrocarbon molecules in liquid and crystalline phases has been extensively studied both experimentally^4^,^5^,^10^,^11^,^12^ and theoretically^13^,^14^,^15^,^16^. According to these studies, high pressure favors breaking of double or triple π-bonds which produces radical species that trigger chain reactions. These HP reactions occur above a pressure threshold and proceed slowly on a timescale of several hours or days. Two approaches have been considered to accelerate these reactions. The first approach consists of increasing the pressure. However, due to the reduction of molecular mobility with increasing pressure, this approach is restricted to monomers in ordered phases in which the polymerization does not require large molecular rearrangements^5^,^17^. Therefore, photoactivation is commonly considered to be the unavoidable approach for accelerating the reaction kinetics and reducing the pressure threshold required to initiate polymerization^18^.

Here, we report that polymerization reactions can be efficiently induced by a new high-pressure-ramp (HPR) process without photochemical or thermal activation. The efficiency of this new approach is demonstrated on the bulk polymerization of the 2-(hydroxyethyl)methacrylate (HEMA) monomer. In addition to its high efficiency, the major advantage of HPR polymerization is the production of long polymer chains in a very short reaction time (few minutes), which is much shorter than that (several hours to days) required for polymerization at static high pressure^4^,^5^,^6^,^10^,^11^,^12^. Due to the large field of application of poly-HEMA (pHEMA) as a highly biocompatible material^19^,^20^, the HPR process leading to the most pure pHEMA without the use of potentially toxic catalysts, is extremely appealing for medical applications or elaboration of photosensitive organic-inorganic hybrid materials^21^,^22^,^23^.

The polymerization of HEMA is typically triggered by radicals produced by thermal or photoexcitation. It proceeds via a classical addition reaction in three steps: initiation, propagation and termination. The proposed HPR process enables to control the first two steps of the sequence. The first step is initiated at pressures greater than 6.5 GPa, at which radicals are formed from excited HEMA monomers. Their reactivity is however hindered by the dense glass-like environment. The second step is completed at pressures less than 2 GPa, at which the sample becomes again liquid, and the radicals are released from their fixed positions at a higher pressure. The process is shown in Fig. 1, in which the diamond anvil cell (DAC) used to apply the pressure is illustrated, along with a photographic image of the recovered sample.

## Results

**Figure 2:**
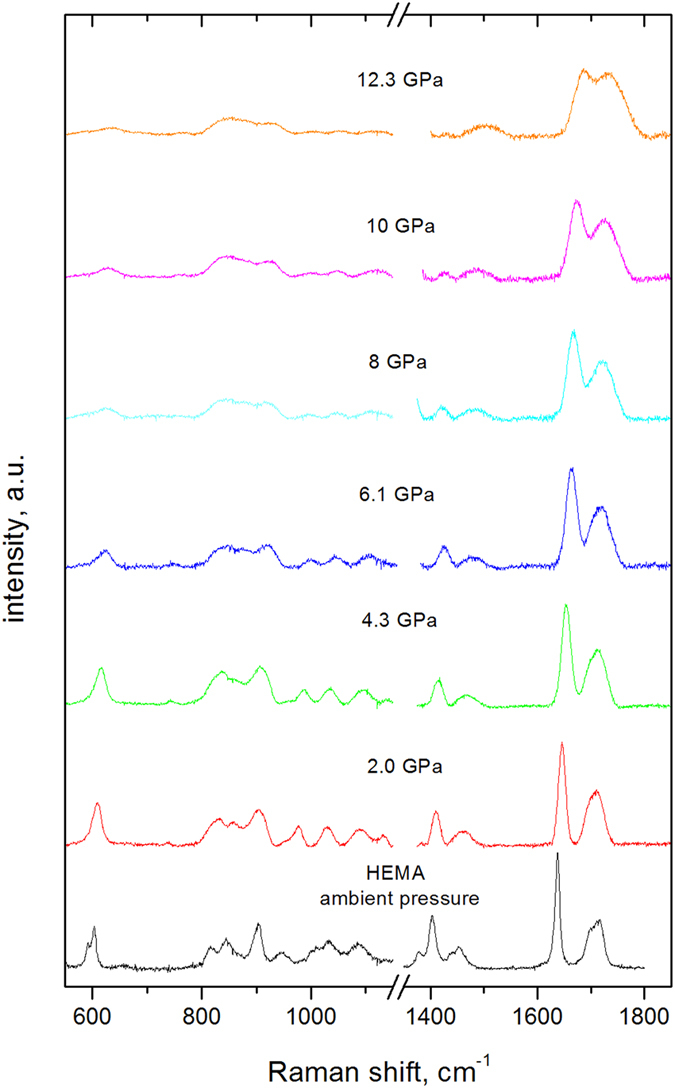
Raman spectra of HEMA upon compression from ambient pressure to 12 GPa. At ambient pressure, four main vibrational bands are observed: the C=C bond at 1407 and 1641 cm^−1^ associated respectively with the C=CH_2_ stretching and C=C aliphatic stretching vibrations, the C–CH bond at 1455 cm^−1^ associated with deformation of C–H group and C=O stretching at 1714 cm^−1^ (see supplementary table 1).

We have used Raman spectroscopy to monitor the evolution of HEMA under increasing pressure (Fig. 2). The energies and assignments of the observed Raman bands are reported in Supplementary Table 1. Because the C=O bond is not involved in the polymerization process, its stretching vibrational band at 1710 cm^−1^ can be used to normalize the intensities of the spectra. These spectra indicate that HEMA does not polymerize when subjected to increasing pressures between 2 and 15 GPa. Indeed, if polymerization occurred, the intensity of the characteristic C=C vibrational modes at 1404 and 1640 cm^−1^ would decrease, and the intensity of the C–CH_2_ deformation mode at 1452 cm^−1^ would increase with the elongation of the polymer chains^6^,^24^. This hypothesis disagrees with our observations for HEMA on compression because the decrease in the C=C band intensity in these series was not correlated with an increase in the C–CH_2_ band intensity. No further modifications of the spectra were observed over a period of several days when HEMA was maintained at a fixed pressure.

When the pressure increases relative intensities of bands related to C–CH_2_ deformation and C=O stretching remain roughly constant. On the other hand, compared to these bands, relative intensities of bands related to C=C (1404 and 1640 cm^−1^) strongly decrease (see supplementary figure 2). We think that this behavior does not only result from changes of Raman polarizabilities. A change in the Raman polarizability requires a significant change in the geometry of HEMA molecule. However, the strength of intramolecular bonds in HEMA, especially of the C=C double bonds, is high enough to resist the compression achieved in our experiments. Indeed, XRD investigation on high pressure behavior of crystalline polyethylene^25^ has shown that directional elastic modulus along the chains of ethylene molecules in the crystalline phases is at least 3500 GPa. The intramolecular incompressibility in the ethylene molecule (H_2_C=CH_2_) should be even higher because the latter contains also a much weaker resistance against deformation between the adjacent molecules in the chain. Accordingly, the maximal pressure of 12.3 GPa achieve in our work is certainly far too low in order to be able to influence significantly geometry and polarizability of the C=C double bond.

We assign the observed modifications of the Raman spectra in the energy range 1400-1800 cm^−1^ to the activation of HEMA monomers by the C=C double bond opening without the formation of polymer chains. The process is reversible, and a rapid opening of the DAC results in the relaxation of excitation, as shown by the Raman spectra of the recovered liquid sample:


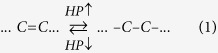


The decrease in the C=C band intensity was due to the thermal population of the T_1_(^3^ππ^*^) triplet state. Indeed, the electron in the π^*^ orbital lengthens the C=C double bond, which acquires a single-bond character^26^,^27^.

The Raman bands of HEMA became stiffer and broader as the pressure increased. Their frequency shifts as a function of pressure are shown in Supplementary Figure 1, and the coefficients 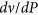 are listed in Supplementary Table 1. The absence of discontinuities in this behavior (i.e., progressive broadening, similar coefficients of linear shift and absence of new vibrational bands) at pressures less than 15 GPa indicates that there is no transition to a hypothetical crystalline phase. Structureless XRD patterns of the compressed samples also support this explanation. In addition, viscosity measurements exhibited an abrupt densification of HEMA at pressures of approximately 2 GPa^6^, which is assigned to the glass transition.

**Figure 3:**
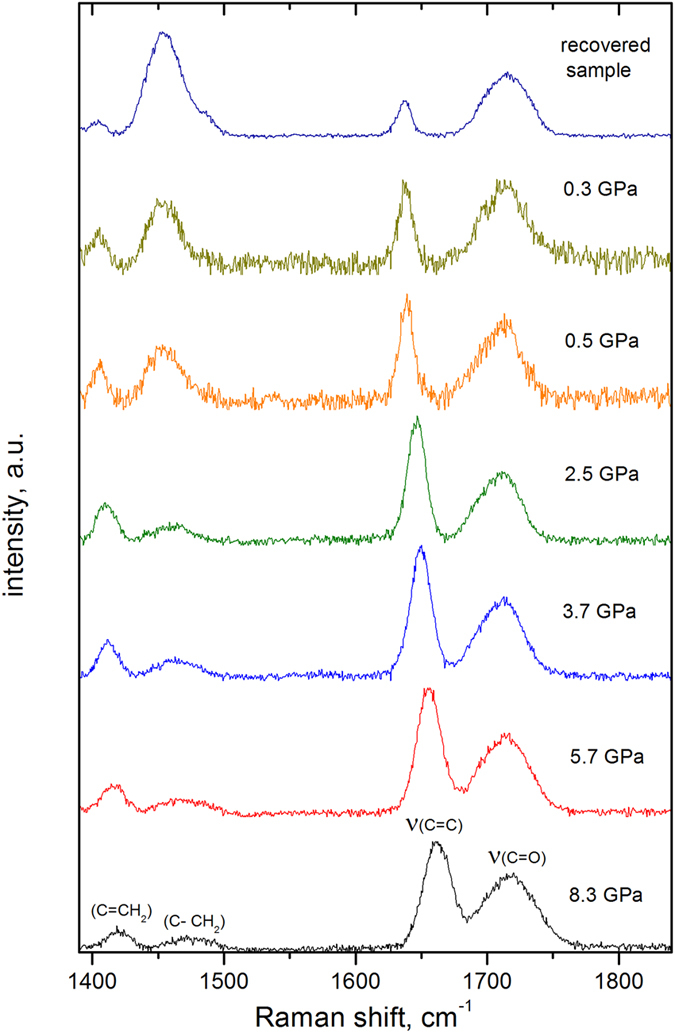
Raman spectra of HEMA at selected pressures during step-by-step decompression from 8.3 GPa to ambient pressure. The sample was maintained at each transient pressure for a few minutes.

According to reaction (Eq. 1), the increase in pressure shifts the equilibrium between the singlet S_0_ and triplet T_1_ states of the HEMA molecule toward the T_1_ state. The reaction of the HEMA(T_1_) molecules was restricted by the steric environmental constraint. However, when the pressure was decreased to less than the critical pressure (*p*_*c*_ = *2* GPa), were the monomers released from their fixed positions in the glass state? Figure 3 shows the Raman spectra of HEMA compressed at 8.3 GPa and then decompressed in a stepwise fashion to atmospheric pressure. As the pressure decreased from 8.3 to 2.5 GPa, the Raman bands exhibited characteristic softening. The relative intensity of the C=C bands increased slightly, indicating S_0_ ← T_1_ relaxation, and the C–C band intensity remained unchanged. In contrast, when the pressure decreased to 0.5 GPa, the C–C band intensity increased substantially, and the C=C intensity decreased, which is a characteristic feature of polymerization propagation due to the formation of covalent single bonds between monomers. Using this pressure ramp the transformation becomes irreversible


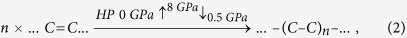


The recovered sample fills the entire volume of the DAC and its Raman spectrum (supplementary Figure.3) exhibits similar to that of pHEMA produced by conventional free-radical polymerization with initiators.

Few HP reactions induced by compression-decompression cycle have been already reported in the case of aromatic molecules like benzene and furan^28^,^29^,^30^. Both molecules start to react in the solid phase at relatively high pressures, respectively 23 and 1O GPa. Nevertheless, the reaction mainly proceeds when the pressure is released through the opening of the aromatic rings. This behavior was assigned to the large rearrangement the molecules must undergo to react. Such movements, prevented at high pressure by the high density of the crystal, become possible under decompression when the crystal structure relaxes to accommodate the bonds reorganization. Eventually, recovered samples based on amorphous hydrogenated carbon compounds (a:C-H) are obtained.

**Figure 4:**
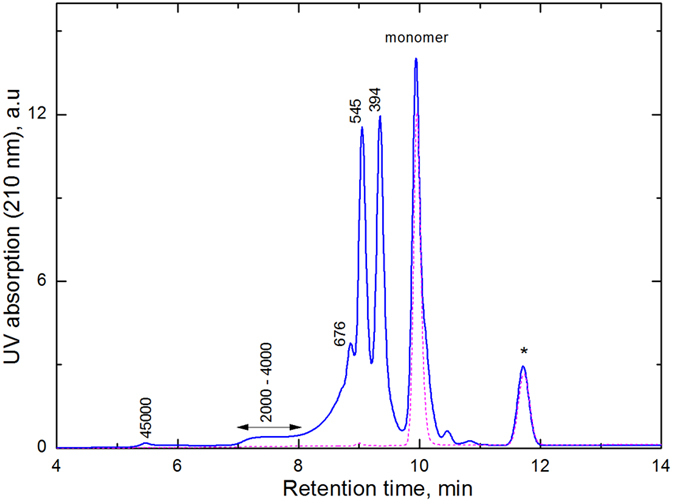
Gel permeation chromatogram of pHEMA synthesized using the HPR process. Molecular weights are reported in g/mol. The chromatogram of liquid HEMA prior to the HP treatment is represented by a dotted line. The *peak corresponds to THF impurities.

The apparent disagreement between the reversible (Eq. 1) and irreversible (Eq. 2) reactions mediated by high pressures indicates a kinetically limited polymerization process. The nature of the active species that survive the pressure ramp and trigger the reactions remains unclear. The recovered samples were analyzed by gel permeation chromatography (GPC) to gain insight into these species. For these and subsequent experiments, the standard experimental protocol was as follows: liquid HEMA was compressed to a pressure *p*_*1*_, then decompressed to *p*_*2*_ and maintained at this pressure for 5 min prior to opening the cell and analyzing the sample. The dissolution of the recovered sample in tetrahydrofuran (THF) for GPC analysis was not complete, indicating the formation of long polymer chains. The chromatograms of liquid HEMA and the sample recovered after the HPR process are shown in Fig. 4. The HEMA monomer (with molecular weight of 130 g/mol) was detected at 9.96 min. The peaks with molecular weights of 394, 545 and 676 g/mol were assigned to oligomers formed by 3, 4 and 5 monomers, respectively. Due to the incomplete solubility of the recovered sample, the relative intensities of the GPC peaks for long polymer chains underestimated their real populations. The shoulder at 7–8 min corresponds to chains with a molecular weight of 2000–4000 g/mol, and the peak at 5 min was due to larger polymers with weights of approximately 45000 g/mol. Therefore, the GPC analysis indicated that the HPR process results in rapid polymerization of HEMA without sacrificing the polymer chain length.

The GPC analysis indicated the remarkable absence of HEMA dimers in the recovered sample, which signifies their preferential consumption during the polymerization. However, this consumption is possible only if these dimers are radical species that trigger the reaction. The HEMA_2_^••^ biradicals can be spontaneously formed in the first step of compression at pressures greater than 2 GPa when HEMA is in glass-like state. These biradicals react later when they leave their fixed positions at pressures less than 2 GPa and a rapid polymerization process takes place promoted by a high concentration of the excited biradicals. Therefore, the final species in the polymerized samples are HEMA and (HEMA)_n_ species with *n* > *2*. The role of excited triplet state biradicals as polymerization initiators has been previously proposed by Lingnau *et al.*^31^,^32^,^33^,^34^ and recently confirmed by quantum chemical calculations^35^ to explain the spontaneous polymerization of methyl methacrylate at high temperatures and ambient pressure. The formation of excited biradical dimers in triplet state has also been suggested to explain the polymerization of small hydrocarbon monomers at high pressures^16^. Our results suggest that polymers could be initiated by biradicals.

**Figure 5:**
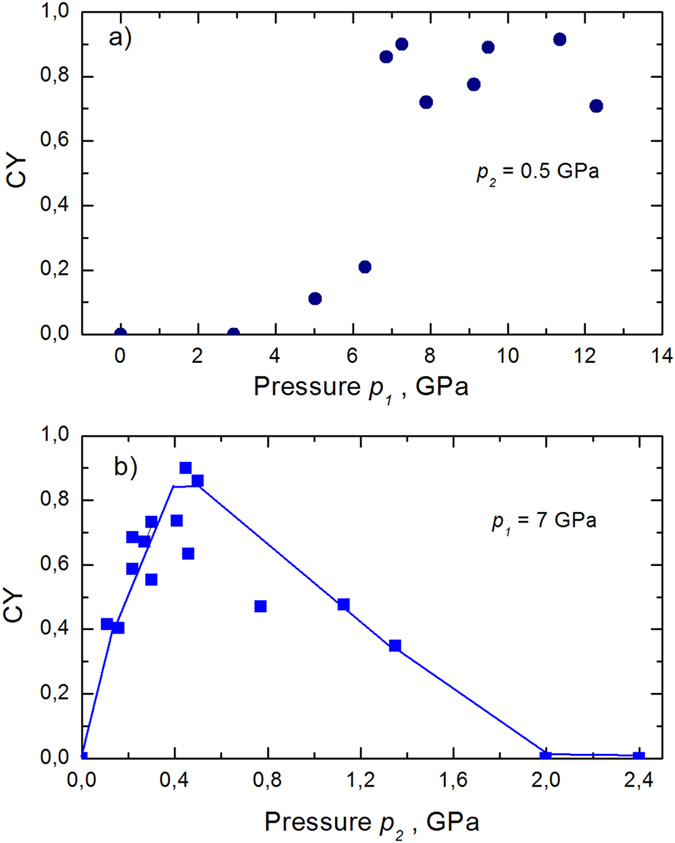
Conversion yield as a function of pressures p_1_ (a) and p_2_ (b) of the two-stage HPR process (decompression pressure p_2_ = 0.5 GPa (a) and compression pressure p_1_ = 7 GPa (b) were used).

The efficiency of the HPR process, which is defined by pressures of the first (*p*_*1*_) and second (*p*_*2*_) stages, was obtained from measurements of the conversion yield (CY) according to^6^:


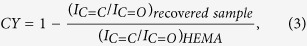


where *I*_*C=C*_ and *I*_*C=O*_ are the integrated intensities of the ν(C=C) and ν (C=O) Raman bands, respectively. The CY as a function of *p*_*1*_ (with fixed *p*_*2*_ = *0.5* GPa) and *p*_*2*_ (with fixed *p*_*1*_ = *7* GPa) are shown in Fig. 5a,b. The CY exhibits a threshold at *p*_*1*_ = *2* GPa and reaches a plateau with a maximum of 90% for pressures higher than 6.5 GPa (Fig. 5a). In the second series (Fig. 5b), the CY strongly increased with the pressure (*p*_*2*_) and reached a maximum at 0.4 GPa. With a further increase in the pressure, the CY decreased to zero at *p*_*2*_ ≥ *2* GPa. In addition, the CY is also zero at atmospheric pressure (*p*_*2*_ = *0.1* MPa). The optimal domain of pressures in the HPR process can be defined as p_1_ ≥ 6.5 GPa and *p*_*2*_ = *0.5* GPa. The polymerization initiation (at *p*_*1*_) and propagation (at *p*_*2*_) kinetics are relatively rapid and complete on the timescale of approximately 1 min.

Interestingly, when hydroquinone, used a holes inhibitor, is added to HEMA the recovered sample, obtained in the conditions *p*_*1*_ = *7* GPa and *p*_*2*_ = *0.5* GPa, exhibits a conversion yield of 15% significantly lower than the 90% measured in absence of hydroquinone. This result suggests that polymerization proceeds through radical way. Nevertheless, the modifications induced by HP on hydroquinone molecule are unknown. In particular, the reactivity of hydroquinone in dense media could be different to that at ambient pressure what would explain the observed residual polymerization (15%). The radical nature of the initiator species produced during the HP compression step was confirmed in additional experiments, where the sample, which was initially compressed at 9.5 GPa, was quenched to atmospheric pressure and opened for a period of time before a pressure of 0.5 GPa was applied. When the experiment was conducted in a glove box (Ar gas, residual O_2_ and H_2_O <0.5 ppm), a solid sample was recovered with a CY of ≈90%. In contrast, the sample remained liquid (non-polymerized) when the same procedure was performed in ambient air. These results indicated that the polymerization initiators disappear in contact with ambient humidity, which is a strong indication of their radical nature.

**Figure 6:**
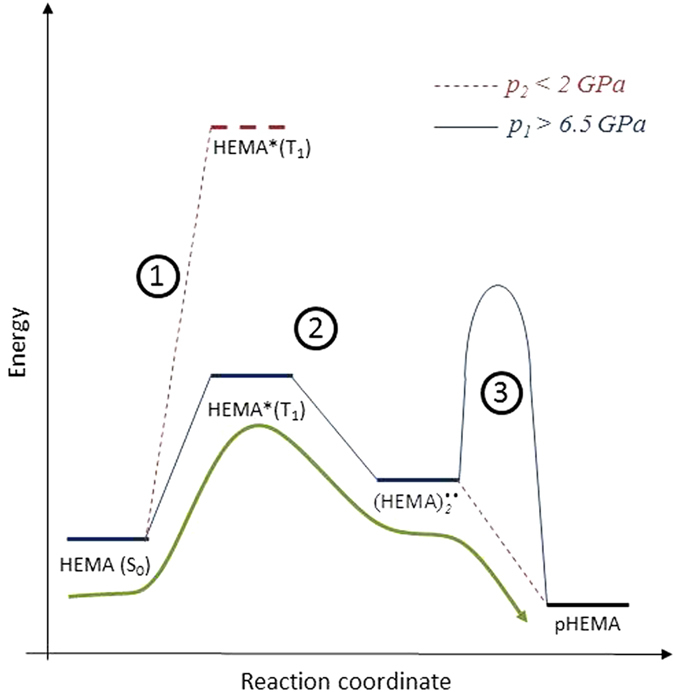
Energy level diagram of HEMA at pressures p_1_ (—) and p_2_ (---) relevant for the HPR process. The energy barrier represents the constraints induced by the environment at a given pressure. The arrow represents the system evolution during polymerization in the HPR process (see the text for more details).

Because HEMA polymerizes without cross-linking, polymerization requires significant mobility of the monomers to accommodate the growing chain. Therefore, the reaction is sensitive to the constraints induced by the environment. The experimental results presented above can be rationalized in the framework of the model described below. Figure 6 shows the evolution of the energetic structure of HEMA along with the constraints induced by the environment for the relevant pressures of the first (*p*_*1*_ ≥ 6.5 GPa) and second (*p*_*2*_ < 2 GPa) stages. The proposed reaction pathway, which is represented by an arrow, is based on the formation of an excited biradical 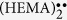
at pressure *p*_*1*_ and the subsequent propagation of the polymer chain at pressure *p*_*2*_. The main features of this mechanism are as follows:

1. The increase in pressure reduces the energy gap between the occupied π(HOMO) and unoccupied π^*^(LUMO) orbitals, enabling population of excited states^13^,^18^,^36^. Therefore, some HEMA molecules are excited in the T_1_(^3^ππ^*^) triplet state, which is characterized by double-bond opening (Eq. 1). The reversible decrease in the C=C bond intensity in the Raman spectra confirmed this conclusion (Fig. 2).
2. At pressures of p_1_ ≥ 6.5 GPa, a favorably oriented excited monomer HEMA(T_1_) reacts with other monomers to form a metastable activated biradical HEMA_2_^••^, which is stable at any pressure but disappears in contact with atmospheric humidity. Dimerization occurs when the intermolecular distance becomes sufficiently short to make the system unstable^13^,^14^,^15^,^16^ . This result was validated experimentally. The reaction of the biradical is prohibited (i) at pressures above 2 GPa due to its steric confinement in the amorphous phase of HEMA and (ii) at low pressures of less than 0.1 GPa due to its limited reactivity.
3. HEMA returns in the liquid state on decompression to pressures *p*_*2*_ less than 2 GPa. The accompanying decrease in the energy barriers (Fig. 6) releases the biradicals from their fixed positions. Therefore, the reactions of HEMA_2_^••^ with monomers are no longer sterically hindered:

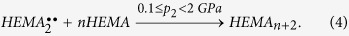


As a result, the formation of all of the oligomeric species except HEMA_2_ is allowed and proceeds rapidly.

Recently, the HP-induced self-polymerization of HEMA at room temperature was observed in a very limited range of pressures from 0.1 to 1.6 GPa^6^. In comparison to this static HP approach, the HPR process increases the CY from 30% to 90% and reduces the reaction time from few weeks to couple of minutes. The higher rate and efficiency of the HPR process can be related to the high concentration of HEMA_2_^••^ biradicals that are formed in the first stage of the process. In contrast, the high energy of the HEMA(T_1_) triplets reduces the probability of their direct thermal excitation at pressures less than 1.6 GPa (see Fig. 6). Although the UV photoexcitation of the HEMA(T_1_) triplet increases the reaction rate^6^, it remains much slower than that of the presently proposed HPR process, that also offers higher efficiency when compared with both the static HP and photoassisted static HP processes.

## Conclusion

The described HPR induced polymerization is a powerful method for controlling polymerization reactions and an appealing alternative to the photochemical activation of chemical reactions at high pressures. In addition, this approach extends the application field of HP-induced polymerization beyond the family of ordered (crystallized) monomers considered up today. The possibility of the rapid and efficient polymerization of HEMA using the HPR process without a catalyst, significantly reinforces the potential for synthesis of highly biocompatible materials. In comparison to the typical radical polymerization methods with thermal or photon activations, the HPR process will reduce the environmental impact of large-scale fabrication. The possibility to separate activation and propagation stages of the polymerization process, will also enable the fabrication of high-purity thin films, organic or hybrid composites and other biocompatible materials.

## Methods

### High pressure experiments

Liquid HEMA (purity >99%, Aldrich) without further purification was loaded into a 150 μm diameter hole drilled in a preindented metal gasket and was compressed in a symmetric diamond anvil cell (DAC). The anvils, which were made of IA type diamonds, were selected due to their low fluorescence in the frequency range of the Raman measurements. Pressure of samples in the DAC was monitored with a precision of ±0.05 GPa using the ruby fluorescence scale. The Raman spectra of the samples compressed at room temperature were measured in the backscattering configuration using a HR800 spectrometer equipped with a Peltier-cooled CCD detector (Horiba Jobin Yvon) with spectral and spatial resolutions of 0.25 cm^−1^ and 5 μm, respectively.

The samples (0.1 mg) for chromatographic analyses were prepared in a Bridgman anvil system^37^. Liquid HEMA was loaded into a 1-mm diameter hole drilled in a stainless steel gasket (thickness 250 μm), and compressed in a symmetrically opposed anvil system. The anvils were made of tungsten carbide sintered with 12% of cobalt (WC + 12% Co) and had the working surface diameter of 3 mm. The pressure on the opposed anvil system was applied by a commercial tensile and compression press (Instron 1195). The samples were compressed up to 7.5 GPa and immediately decompressed down to 0.5 GPa, which was maintained for 10 minutes. As a result, solid polymerized samples (diameter 800 μm, thickness 120 μm) were obtained.

### Gel permeation chromatography

The molecular weight and polydispersity of the polymeric chains were measured using gel permeation chromatography (GPC). The Dionex Ultimate 3000 GPC apparatus was equipped with a Polymer Laboratories MesoPore 3-μm column (300*7.5 mm) in an isothermal oven (35 °C) in series with a UV-visible detector (detection wavelength λ = 210 nm). The eluent flow rate (HPLC grade tetrahydrofuran, THF) was set to 1.0 mL/min. Particular attention was paid to the calibration of the column for low molecular weights (100–1000 g/mol). The calibration was performed using a set of polystyrene standards (EasiCal PS-2 Polymer Laboratories, Mp = 580–20650 g/mol range) with a very narrow polydispersity, including ethylene glycol dimethacrylate (CAS: 97-90-5, molecular weight 198.22 g/mol) and lauryl methacrylate (CAS: 142-90-5, molecular weight 254.41 g/mol). The polymerized samples were dissolved in THF in an ultrasonic bath for two hours. Each analysis solution (1 mg polymer dissolved in 2 mL THF) was introduced via a manual injector loop (volume injected 20 μL). Data acquisition and analysis were performed with Chromeleon V 6.80 software supplied by Dionex.

### XRD diffraction

The X-ray diffraction measurements were obtained on the XRD1 beamline^38^ of the ELETTRA synchrotron with monochromatized radiation of λ = 0.6889(1) Å collimated with a 30-μm pinhole. The XRD patterns were collected with a 2D detector Dectris Pilatus 2M (1475 × 1679 pixels, pixel size 172 × 172 μm^2^) located 170 mm from a sample compressed in a Boehler-Almax plate DAC^39^. The latter was mounted on a Huber Kappa Goniometer and oscillated during the data collection by ±2°.

## Additional Information

**How to cite this article**: Evlyukhin, E. *et al.* A New Route for High-Purity Organic Materials: High-Pressure-Ramp-Induced Ultrafast Polymerization of 2-(Hydroxyethyl)Methacrylate. *Sci. Rep.* **5**, 18244; doi: 10.1038/srep18244 (2015).

## Supplementary Material

Supplementary Information

## Figures and Tables

**Figure 1:**
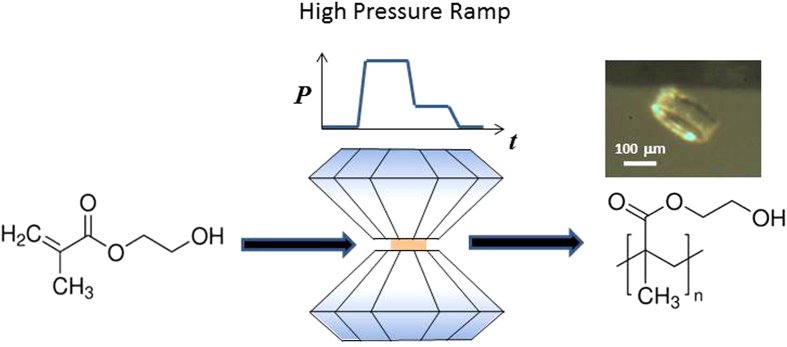
Scheme for HEMA polymerization using the HPR process. The photographic image shows a typical recovered sample.
