# Supplementary material for: A New Route for High-Purity Organic Materials: High-Pressure-Ramp-Induced Ultrafast Polymerization of 2-(Hydroxyethyl)Methacrylate
Source: Sci Rep. 2015 Dec 16;5:18244. doi: 10.1038/srep18244 (PMC4680890; doi:10.1038/srep18244)
Supplement: Supplementary Information [file srep18244-s1.pdf]

A New Route for High-Purity Organic Materials: High-Pressure-Ramp-Induced Ultrafast Polymerization of 2-(Hydroxyethyl)Methacrylate

E. Evlyukhin,[a] *L. Museur,*[a*] *M. Traore,*[b] *C. Perruchot* [c] *, A. Zerr,*[b] *A. Kanaev*[b]

a) Laboratoire de Physique des Lasers - LPL, CNRS, Université Paris 13, Sorbonne Paris Cité, 93430 Villetaneuse, France.

b) Laboratoire des Sciences des Procédés et des Matériaux - LSPM, CNRS, Université Paris 13, Sorbonne Paris Cité, 93430 Villetaneuse, France.

c) Laboratoire Interfaces Traitements Organisation et Dynamique des Systèmes - ITODYS CNRS, Université Paris Diderot Paris 7, Sorbonne Paris Cité, 75205 Paris Cedex 13, France

Raman shift (cm^{-1})	dv/dP (cm^{-1}/GPa)	Assignment
600	Non-linear	$\nu(CCO)_{sym}$ [1] or $\delta(O-C=O)$ [2]
816	2.7	$\tau(C=C)$ [3], $\nu(COC)_{sym}$ [1], $\omega(C=O)$ [2] or $\nu(C-CH_3)$ [4]
845	3.1	$\gamma(CH_2)$ [1] or $\nu(C-C)$ [2, 3]
900	2.4	$\nu(CO)$ [1]
947	Non-linear	deformation localized on OCH_2CH_2OH part of HEMA molecule
1023	2.5	$\nu(CC)$ [1]
1085	3.1	$\gamma(CH_2)$ [2, 3]
1404	2.3	$\delta(=CH_2)$ [2, 3] or $\nu(C=CH_2)$ [5]
1455	3.6	$(C-CH_2)$ deformation [6, 7]
1640	3.6	$\nu(C=C)$ [1-3]
1705		$\nu(C=O)$ [1-3] free
1714		$\nu(C=O)$ [1-3] H-bonded

Supplementary Table 1 Energies and assignments of the bands observed in the Raman spectra of HEMA at ambient pressure. Here ν is bond stretching, δ bending, ω wagging, γ rocking and τ torsion

1. Bertoluzza, A., et al., *Applications of Raman spectroscopy to the ophthalmological field : Raman spectra of soft contact lenses made of poly-2-hydroxyethylmethacrylate (PHEMA).* Journal of Molecular Structure, 1986. **143**(0): p. 469-472.
2. Faria, M.D.G., J. Teixeira-Dias, and R. Fausto, *Conformational stability for methyl acrylate: a vibrational spectroscopic and ab initio MO study.* Vibrational spectroscopy, 1991. **2**(1): p. 43-60.
3. Kulbida, A., et al., *Rotational isomerism in acrylic acid. A combined matrix-isolated IR, Raman and ab initio molecular orbital study.* J. Chem. Soc., Faraday Trans., 1995. **91**(11): p. 1571-1585.
4. Arenas, J.F., et al., *SERS of acrylic acid and methyl derivatives on silver sols.* Journal of Raman Spectroscopy, 1998. **29**(7): p. 585-591.
5. Mabilleau, G., et al., *Polymerization of 2-(hydroxyethyl)methacrylate by two different initiator/accelerator systems: a Raman spectroscopic monitoring.* Journal of Raman Spectroscopy, 2008. **39**(7): p. 767-771.
6. Colthup, N., *Introduction to infrared and Raman spectroscopy*. 2012: Elsevier.
7. Lambert, J.B., et al., *Introduction to organic spectroscopy*. 1987: Macmillan Publishing Company.

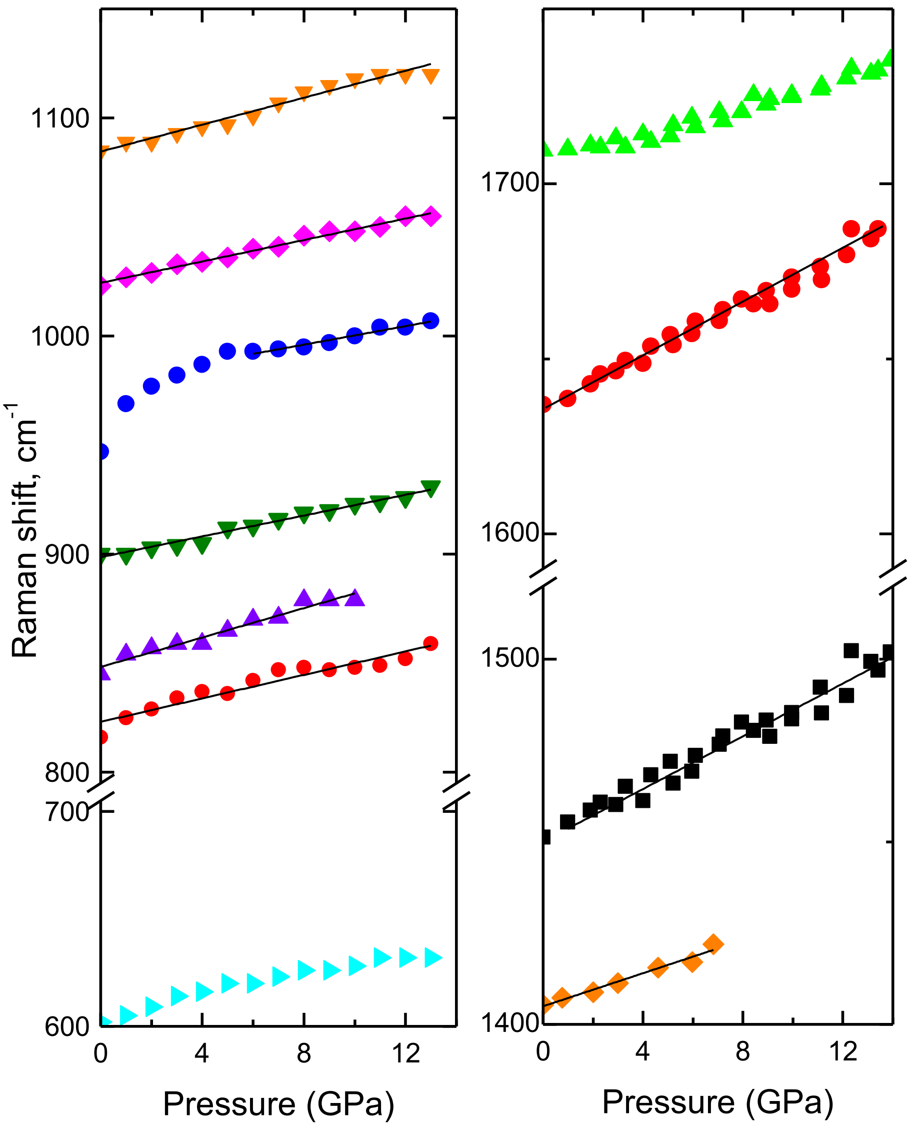

Supplementary Figure 1: Raman shift of HEMA as a function of pressure on compression. Solid straight lines are linear fits of the data.

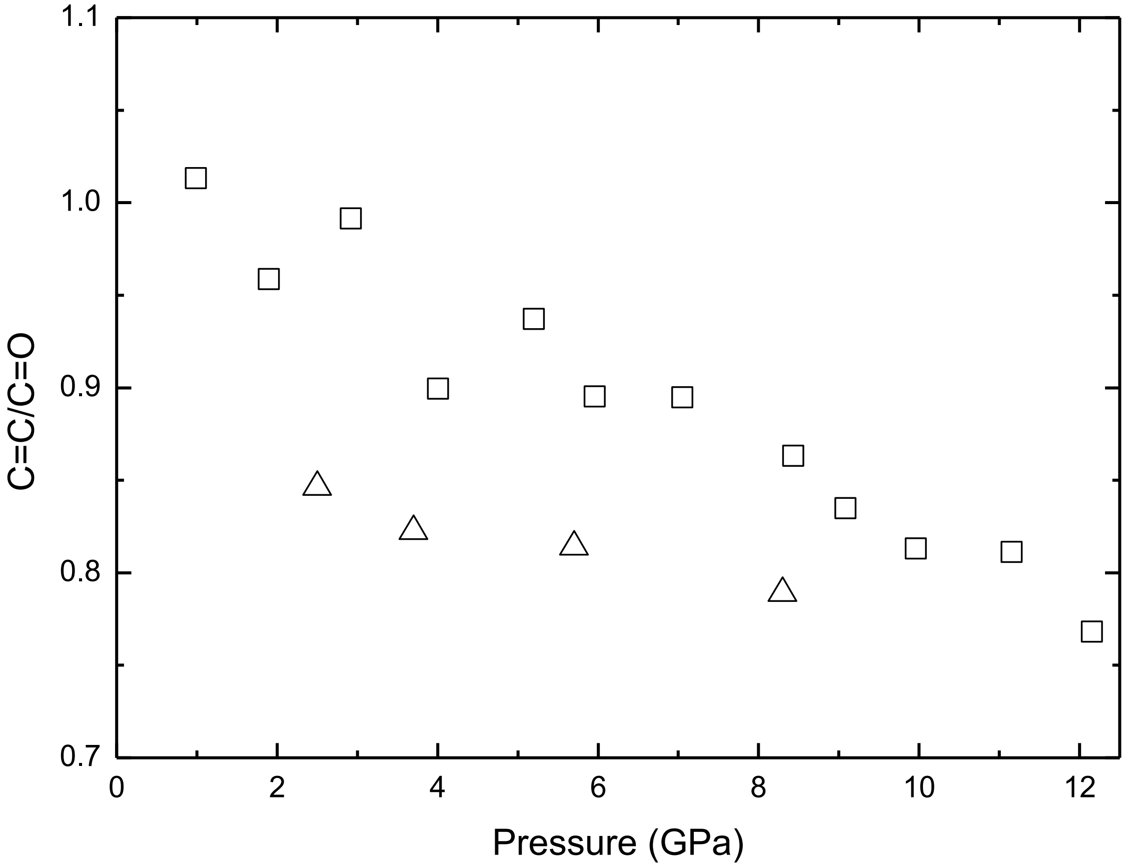

Supplementary Figure 2: Intensity ratio of Raman bands associated with the stretching modes of C=C (1640 cm^{-1}) and C=0 (1710 cm^{-1}) bonds as function of pressure under compression (□) and decompression (Δ). The intensity is calculated from the area of the Raman bands

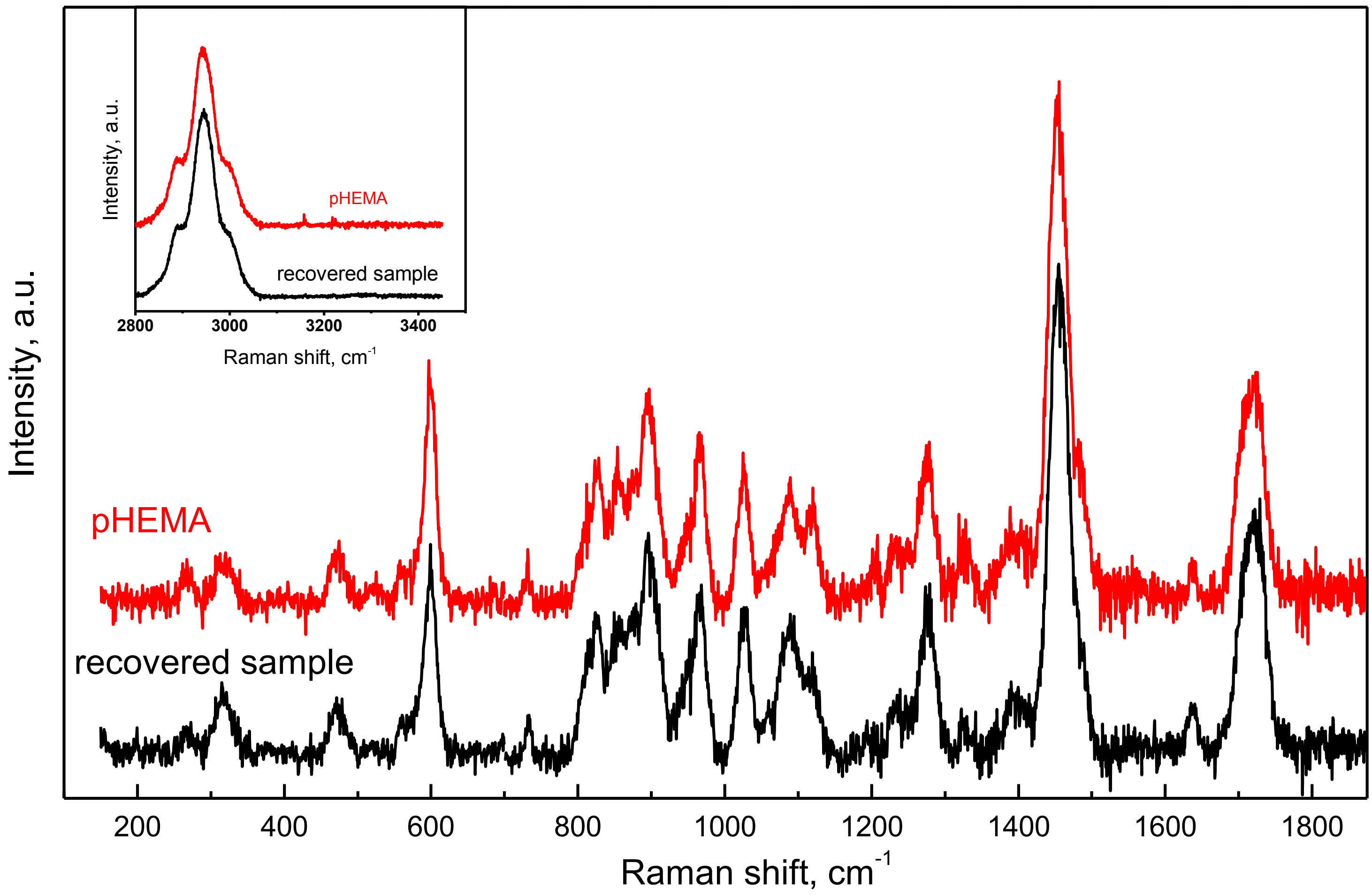

Supplementary Figure 3: Raman spectra of (i) pHEMA polymerized with thermal initiators (red), and (ii) freshly recovered sample after HPR process (black)
